# Differential Diagnosis of Rare Subtypes of Progressive Supranuclear Palsy and PSP-Like Syndromes—Infrequent Manifestations of the Most Common Form of Atypical Parkinsonism

**DOI:** 10.3389/fnagi.2022.804385

**Published:** 2022-02-09

**Authors:** Patrycja Krzosek, Natalia Madetko, Anna Migda, Bartosz Migda, Dominika Jaguś, Piotr Alster

**Affiliations:** ^1^Students’ Scientific Association of the Department of Neurology, Medical University of Warsaw, Warsaw, Poland; ^2^Department of Neurology, Medical University of Warsaw, Warsaw, Poland; ^3^Department of Internal Medicine and Endocrinology, Medical University of Warsaw, Warsaw, Poland; ^4^Diagnostic Ultrasound Lab, Department of Pediatric Radiology, Medical Faculty, Medical University of Warsaw, Warsaw, Poland

**Keywords:** progressive supranuclear palsy, subtypes, variants, PSP-like syndrome, differentiation

## Abstract

Presently, there is increasing interest in rare PSP (progressive supranuclear palsy) variants, including PSP-PGF (PSP-progressive gait freezing), PSP-PI (PSP-postural instability), PSP-OM (PSP-ocular motor dysfunction), PSP-C (PSP-predominant cerebellar ataxia), PSP-CBS (PSP-corticobasal syndrome), PSP-SL (PSP-speech/language disorders), and PSP-PLS (PSP-primary lateral sclerosis). Diagnosis of these subtypes is usually based on clinical symptoms, thus thorough examination with anamnesis remains a major challenge for clinicians. The individual phenotypes often show great similarity to various neurodegenerative diseases and other genetic, autoimmune, or infectious disorders, manifesting as PSP-mimicking syndromes. At the current stage of knowledge, it is not possible to isolate a specific marker to make a definite ante-mortem diagnosis. The purpose of this review is to discuss recent developments in rare PSP phenotypes and PSP-like syndromes.

## Introduction

Progressive supranuclear palsy (PSP) has been known for more than half a century, but most of the reports are on PSP-Richardson syndrome (PSP-RS) and PSP-P (PSP-Predominant Parkinsonism) (Steele et al., 1964; Williams et al., 2005a). Growing interest is now associated with less common PSP phenotypes, including PSP-PGF, PSP-PI, PSP-OM, PSP-C, PSP-F, PSP-CBS, PSP-SL, and PSP-PLS. Most of them are included in The Movement Disorders Society (MDS) criteria, introduced in 2017 (Höglinger et al., 2017). This results in a higher sensitivity in the diagnosis of PSP compared with the National Institute of Neurological Disorders and Stroke and the Society for PSP (NINDS-SPSP) criteria issued in 1996 (87.9% for MDS criteria versus 45.5% for NINDS-SPSP criteria) (Litvan et al., 1996; Ali et al., 2019). The pathology of PSP is a 4-repeat tauopathy, and its phenotypic diversity is related to the accumulation of tau protein in different brain areas (Rösler et al., 2019; Kovacs et al., 2020). On the other hand, many other diseases may clinically resemble PSP. Effective *in vivo* studies of rare PSP phenotypes and PSP-mimicking syndromes are not available. This paper presents an overview and differential diagnosis of these disorders.

## Progressive Supranuclear Palsy-Subcortical

### Progressive Supranuclear Palsy-Progressive Gait Freezing

The main symptom of PSP-PGF is a gradual increase in difficulty with gait initiation that is levodopa-resistant (Williams et al., 2007; Höglinger et al., 2017). Imaging studies show deterioration limited to the basal ganglia, including atrophy in volumetric magnetic resonance imaging (MRI) and increased tracer uptake in 18F-flortaucipir positron emission tomography (PET) (Schonhaut et al., 2017; Whitwell et al., 2020).

PSP-PGF most often causes difficulties in the differential diagnosis with PSP-RS, Parkinson’s disease (PD), and vascular Parkinsonism. Analysis of the rate of progression of symptoms may be useful in distinguishing between the aforementioned diseases. Early supranuclear gaze palsy, particularly combined with advanced executive dysfunction, is usually associated with PSP-RS; whereas, in PSP-PGF, oculomotor dysfunction often appears later, even about 9 years after the onset of the first symptoms (Williams et al., 2007; Respondek et al., 2014; Hong et al., 2015; Lee et al., 2018; Jabbari et al., 2020). Freezing of gait (FoG) is an early symptom in PSP-PGF; whereas, in PD, it generally occurs in late stages, and it is susceptible to levodopa and coexists with slowness of movement, tremor, and rigidity (Factor, 2008; Owens et al., 2016; Höglinger et al., 2017). Pons to midbrain area ratio (P/M ratio) differs among PSP-PGF, PSP-RS, and PD, with the highest ratio value for PSP-RS, the lowest for PD, and at an intermediate ratio for PSP-PGF (Nakahara et al., 2019). However, the precision of differentiation estimated at about 70% based on the P/M ratio is insufficient (Nakahara et al., 2019). Pyramidal tract dysfunction, ischemic changes on imaging studies, and a history of stroke may lead the diagnosis to vascular Parkinsonism, because these features are generally not characteristic of PSP-PGF (Williams et al., 2007).

### Progressive Supranuclear Palsy-Postural Instability

Progressive Supranuclear Palsy-Postural Instability (PSP-PI) is associated with predominant falls and balance problems (Kurz et al., 2016; Höglinger et al., 2017). Some studies present a correlation between the damage to the indirect locomotor pathway with its connections to the pedunculopontine nucleus and postural instability in PSP patients (Brown et al., 2020). Decreased 18F-FDG-PET metabolism is observed in the structures involved in this circuit compared to the group of healthy controls; however, increased metabolism is found within the precentral gyrus (Zwergal et al., 2011, 2013). Precuneus, sensorimotor gyrus, frontal cortex, and parietal cortex present an increased uptake of 18F-THK5351 in PSP patients with unjustified, repetitive falls within 3 years [*P1* feature according to the MDS criteria (Höglinger et al., 2017) compared to the patients without *P1* feature (Hsu et al., 2020)].

PSP-PI may cause difficulties in the differential diagnosis with PSP-RS. One study (Respondek et al., 2014) evaluated symptoms progression in PSP variants. Among the 18 patients with PSP-PI, there was no oculomotor dysfunction during the first year of the disease. In a group of 24 patients with PSP-RS, this symptom was common, affecting approximately 40% of patients. Therefore, the appearance of supranuclear gaze palsy in the first year of the disease may argue against the PSP-PI, although the diagnosis should not be based solely on this one premise.

### Progressive Supranuclear Palsy-Ocular Motor Dysfunction

Progressive Supranuclear Palsy-Ocular Motor Dysfunction (PSP-OM) is associated with predominant oculomotor dysfunction (Höglinger et al., 2017). There is no imaging data in PSP-OM, but one study presents a correlation between the degree of decreased fractional anisotropy (FA) index in the midbrain in MRI and the severity of abnormal vertical eye movements in PSP (Quattrone et al., 2019). In PSP patients with supranuclear gaze palsy [feature O1 according to the MDS criteria (Höglinger et al., 2017)], midbrain damage is greater compared to those with slow velocity of saccades [feature O2 according to the MDS criteria (Höglinger et al., 2017; Quattrone et al., 2019)]. It is also consistent with another PET study that shows an increased 18F-THK5351 uptake in midbrain, raphe nucleus, and red nucleus in PSP patients with ocular motor dysfunction compared with the group without oculomotor abnormalities (Hsu et al., 2020). The anterior cingulate cortex may also be involved in the pathogenesis of downward gaze palsy in PSP, since one study presents bilateral hypometabolism in the anterior cingulate cortex in 18-FDG-PET studies in these cases (Amtage et al., 2014).

The differential diagnosis mainly includes PSP-RS. In one study (Respondek et al., 2014), among 7 patients with PSP-OM, no falls were documented within the first year of the disease. In a group of 24 patients with PSP-RS, this symptom was common, affecting approximately 80% of patients. Therefore, the appearance of falls in the first year of the disease may argue against PSP-OM. This can be a valuable clue, but should not be the single determining factor in the diagnosis.

### Progressive Supranuclear Palsy-Predominant Cerebellar Ataxia

Progressive Supranuclear Palsy-Predominant Cerebellar Ataxia (PSP-C) appears to be a common subtype of PSP only in Asia, but the exact factors contributing to the disproportionate occurrence of this variant worldwide have not yet been determined (Kanazawa et al., 2009; Koga et al., 2016; Ando et al., 2020). Precise diagnostic criteria for PSP-C are not established due to its rarity (Höglinger et al., 2017). Falls are not a differentiating factor with other subtypes, but they occur in almost all patients with PSP-C (Ando et al., 2020). There is a case of one patient with advanced-stage PSP-C with widening of the pontocerebellar cistern in MRI (Kanazawa et al., 2012). To date, early-stage PSP-C has been documented in PET studies using [18F]PM-PBB3 also in only one patient (Ishizuchi et al., 2021). Increased tracer uptake was found in the midbrain, subthalamic nucleus, and dentate nucleus (Ishizuchi et al., 2021).

PSP-C may cause difficulties in the differential diagnosis with multiple system atrophy-cerebellar type (MSA-C) and with genetic PSP-like syndromes (Stamelou et al., 2013; Koga et al., 2015). PSP-C is usually associated with later disease onset than MSA-C (approximately 68.8 years versus 58.3 years) and the lack of autonomic dysfunction (Kanazawa et al., 2013). Suspicion of genetic PSP-like syndrome with ataxia should be aroused primarily by a rapid and abrupt course and uncharacteristic neurological (stimulus-sensitive myoclonus, migraine, cataplexy, neuropathy, seizures) and non-neurological symptoms (splenomegaly, psychiatric symptoms, hearing loss) (Stamelou et al., 2013). Differential diagnosis should include familial Creutzfeld Jacob disease (CJD), mitochondrial diseases (including polymerase-gamma mutations), adult- onset Niemann Pick disease type C (NPC), and spinocerebellar ataxia type 2 and type 3 (Stamelou et al., 2013).

## Progressive Supranuclear Palsy-Cortical

### Progressive Supranuclear Palsy-Corticobasal Syndrome

Progressive Supranuclear Palsy-Corticobasal Syndrome (PSP-CBS) is suggested by the overlapping of impaired limb motor function with apraxia, alien limb syndrome, and the disorder of cortical sensory function (Ling et al., 2014; Höglinger et al., 2017). Volumetric MRI can show asymmetric changes in the supplemental motor and premotor area of the cortex or prefrontal cortex in addition to atrophy of the basal nuclei, midbrain, and superior cerebellar peduncle (Whitwell et al., 2010, 2020). Asymmetric reduced 18F-FDG uptake in PET, compared to the group of healthy controls, was also found in the caudate nucleus, the anterior and middle area of cingulate gyrus, middle frontal gyrus and precentral gyrus (Pardini et al., 2019).

PSP-CBS may cause difficulties in the differential diagnosis with Alzheimer’s disease (AD), frontotemporal lobar degeneration with TAR-DNA binding protein (FTLD-TDP), and corticobasal degeneration (CBD) (Whitwell et al., 2010; Coughlin and Litvan, 2020). Analysis of phosphorylated tau protein and amyloid-β1-42 levels in cerebrospinal fluid (CSF) and an amyloid PET scan may be necessary to rule out AD as a major cause of CBS (Höglinger et al., 2017). Myoclonus is not a differentiating factor with other diseases, but it often occurs in patients with CBS-AD (Hu et al., 2009; Shelley et al., 2009; Hassan et al., 2011; Sakae et al., 2019). CBS-AD patients shows as an asymmetric decreased tracer uptake in 18F-FDG-PET and asymmetric atrophy in the MRI in the temporo-parietal areas as opposed to the changes in the PSP-CBS described above (Whitwell et al., 2010; Pardini et al., 2019). On the other hand, in CBS-TDP, atrophy is more pronounced in the prefrontal areas (Whitwell et al., 2010). Genetic testing can be used to rule out the presence of mutations suggestive of FTLD, regarding mainly GRN gene (Arienti et al., 2021). Differential diagnosis of PSP and CBD based on clinical presentation is extremely difficult, so PSP-CBS is generally classified as “probable 4R-tauopathy” (Ali and Josephs, 2017; Höglinger et al., 2017).

### Progressive Supranuclear Palsy-Frontal

Progressive Supranuclear Palsy-Frontal (PSP-F) is generally associated with frontal lobe damage resulting in behavioural and cognitive impairment (Höglinger et al., 2017). In volumetric MRI, the prefrontal cortex is affected, along with the midbrain, basal ganglia, and superior cerebellar peduncle (Whitwell et al., 2020). One study shows a correlation between the deficit of executive functions in PSP and the reduced perfusion of the posterior area of the midcingulate cortex in technetium-99m-hexamethyl-propylenamine-oxime single photon emission computed tomography (99mTc-HMPAO SPECT) (Chiu et al., 2012). Patients included in the study (Chiu et al., 2012) were diagnosed as PSP-RS based on NINDS-SPSP criteria.

Alzheimer’s disease (AD), psychiatric disorders, FTLD-TDP, and frontotemporal lobar degeneration with tau pathology (FTLD-tau) may cause problems in the differential diagnosis of PSP-F (Seeley, 2019; Coughlin and Litvan, 2020). Alzheimer’s disease should be ruled out using CSF biomarkers or amyloid PET (as in the CBS-AD described above) (Seeley, 2019). Low level of neurofilament light chains (Nfl) in cerebrospinal fluid excludes neurodegeneration with a high likelihood; therefore, it may probably be useful in differentiating PSP-F with psychiatric disorders (Vijverberg et al., 2017; Katisko et al., 2019). Genetic causes of FTLD, including MAPT, PGRN and C9orf72 genes, should be excluded; C9orf72 is particularly often related to behavioural variant of frontotemporal dementia, with the presence of motor neuron disease symptoms, which are absent in PSP-F (Rademakers et al., 2012; Pan and Chen, 2013; Seeley, 2019). Cognitive problems in frontotemporal dementia with tau pathology was associated with low perfusion within the anterior midcingulate cortex in 99mTc-HMPAO SPECT in one study (Chiu et al., 2012), as opposed to the PSP changes described above (Vogt, 2016).

### Progressive Supranuclear Palsy-Speech/Language Disorders

Even more than 50% of patients with isolated non-fluent/agrammatic primary progressive aphasia (nfPPA) or progressive apraxia of speech (AoS) develops other symptoms that could lead to a diagnosis of PSP-SL in subsequent years (Rohrer et al., 2010; Whitwell et al., 2019). Progression correlates with the advance of the patient’s age, atrophy of the midbrain, and it is more common in patients with AoS (Whitwell et al., 2019). According to Botha and Josephs (2019), in AoS “*the dorsolateral premotor cortex, motor cortex proper, and supplementary motor areas”* can be affected, while nfPPA is often related to the anterior involvement of the Sylvian fissure regions and opercular regions of the dominant hemisphere. This is reflected in the volumetric MRI findings with the atrophy of the motor cortex and premotor cortex, and with brainstem structures involved to a lesser extent (Whitwell et al., 2020). PET studies using 18F-flortaucipir follow the same pattern with increased tracer uptake in cortical areas (Whitwell et al., 2020). Similarly, there is a case of one patient with PSP and non-fluent progressive aphasia with predominant increased 18F-THK5351 uptake in cortical frontal areas in PET studies (Brendel et al., 2018). NfPPA in PSP in 18F-FDG-PET studies can be correlated with decreased metabolism in the area of the left frontal lobe (including dorsolateral and medial areas) (Roh et al., 2010; Meyer et al., 2017).

PSP-SL causes difficulties in the differential diagnosis with FTLD-tau, FTLD-TDP, AD, and other PSP subtypes (Höglinger et al., 2017; Hofmann et al., 2019). FTLD-tau group account for the majority of all causes of nfPPA (Spinelli et al., 2017). Alzheimer’s disease pathology should be excluded by amyloid PET and CSF biomarkers (as in the CBS-AD described above) (Perini et al., 2019). The fractional anisotropy (FA) index, which assesses the integrity of the white matter, is significantly lower in patients with PSP-SL compared to PSP-RS and PSP-P within the body of the corpus callosum (Lope-Piedrafita, 2018; Whitwell et al., 2021).

### Progressive Supranuclear Palsy-Primary Lateral Sclerosis

Reports concerning PSP-PLS are non-specific, and this variant is not included in the MDS criteria (Höglinger et al., 2017). The presence of symptoms of upper motor neuron damage is a predominant element in the clinical presentation of this phenotype, while it is possible, but not necessary, to find features typical for PSP, including the most characteristic ones—falls and downward gaze palsy (Josephs et al., 2006; Nagao et al., 2012; King et al., 2013). Data on PSP-PLS imaging are limited. In neuropathological studies, an unusual distribution of tau accumulation is noted, with damage concerning mainly the motor cortex area, while relevant involvement of basal nuclei and brainstem is not a constant feature (Josephs et al., 2006; Nagao et al., 2012; King et al., 2013). Damage to the corticospinal tracts is also common (Josephs et al., 2006; Nagao et al., 2012; King et al., 2013).

With regard to the very limited reports on PSP-PLS, the diagnosis of this subtype is extremely difficult. Reduced FA in MRI-DTI in the superior cerebellar peduncle and corticospinal tracts can be a valuable diagnostic clue, as PLS patients with overlapping of PSP features can show damage not only to the corticospinal tract, but also to the superior cerebellar peduncle (Coon et al., 2012).

**FIGURE 1:**
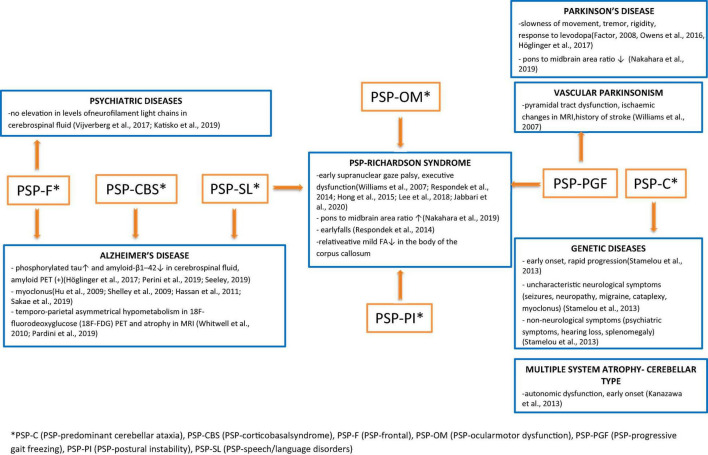
Summary of the differential diagnosis of rare Progressive supranuclear palsy (PSP) subtypes with the indication of uncharacteristic features that lead to deeper diagnostics in the direction of specific disease entities.

A summary of the differential diagnosis of rare variants of PSP is shown in Figure 1.

## Progressive Supranuclear Palsy-Like Syndromes

### Genetic Progressive Supranuclear Palsy

Progressive supranuclear palsy (PSP) can be associated with MAPT mutations (Im et al., 2015). Early onset of the disease, even before the age of 45, suggest a MAPT mutation, as sporadic PSP usually develops the first symptoms at the age of 63–65 (Golbe, 2014; Levin et al., 2016; Wen et al., 2021). The presence of neurodegenerative diseases in family history further increases the likelihood of genetic PSP (Wen et al., 2021). The association of LRRK2 mutations with PSP is still uncertain due to inconsistent study results, but according to some authors, it is considered an extremely rare cause of PSP (Im et al., 2015; Sanchez-Contreras et al., 2017; Wen et al., 2021).

Various genetic diseases can mimic PSP in their clinical picture, and these are called PSP-like syndromes (Stamelou et al., 2013; Wen et al., 2021). Most of these are adverted in other subsections (genetic forms of FTLD, familial CJD, adult-onset NPC, mitochondrial diseases, spinocerebellar ataxia type 2 and type 3), but Perry syndrome and Kukor-Rakeb disease are also worth mentioning (Stamelou et al., 2013). Perry syndrome associated with a DNCT1 mutation can be related to a younger age of onset (approximately 56 years of age) and often with respiratory depression that are not typically found in the sporadic PSP (Stamelou et al., 2013; Höglinger et al., 2017; Barreto et al., 2021; Wen et al., 2021). On the other hand, Kufor-Rakeb syndrome (ATP13A2 mutations) is unique, both for its unusually early onset in young adults and for its response to levodopa treatment (Williams et al., 2005b; Stamelou et al., 2013; Im et al., 2015).

## Paraneoplastic and Autoimmune Progressive Supranuclear Palsy

Progressive supranuclear palsy-like phenotype is potentially related to paraneoplastic syndromes, due to its demonstrated association with anti-Ma1 and anti-Ma2, anti-Hu, anti-CRMP5, and anti-Ri onconeuronal antibodies (Adams et al., 2011; Dash et al., 2016; Ohyagi et al., 2017; Takkar et al., 2020). The latter appear to be frequently correlated with breast cancer (Simard et al., 2020; Takkar et al., 2020). Other neoplasms associated with the syndrome include B-cell lymphoma, small cell lung carcinoma, and tonsil carcinoma (Tan et al., 2005; Adams et al., 2011; Dash et al., 2016).

Progressive supranuclear palsy-like syndrome may be also related to an autoimmune process (Balint et al., 2018). The dynamics of the development of symptoms in autoimmune PSP is different than in neuropathological PSP with the coexistence of atypical features (Table 1) (Hierro et al., 2020; González-Ávila et al., 2021). The association of anti-LGI1, anti-IgLON5, and anti-DPPX antibodies has been demonstrated (Tobin et al., 2014; Gaig et al., 2017; Hierro et al., 2020), but many neuronal antibodies responsible for described phenotype have not yet been characterised (Kannoth et al., 2016). Of particular interest are cases of anti-IgLON5 disease with PSP- like presentation. In both anti-IgLON5 disease and PSP, abnormal vertical eye movements are found; however, upward gaze palsy can predominate in anti-IgLON5 disease, and mainly downward gaze abnormalities are observed in PSP (González-Ávila et al., 2021). The disease can last longer than sporadic PSP, and atypical symptoms may occur (Table 1) (Golbe, 2014; Levin et al., 2016; Gaig et al., 2017). On the other hand, the presence anti-LGI-1 antibodies may be associated with rapid development of symptoms (within months) and clinical improvement following the introduction of immunotherapy (Kannoth et al., 2016; Hierro et al., 2020).

**TABLE 1:** Comparison of paraneoplastic, autoimmune and vascular Progressive supranuclear palsy (PSP) with neurodegenerative PSP.

	Neurodegenerative PSP	Paraneoplastic PSP	Autoimmune PSP	Vascular PSP	Infectious PSP
Disease course	Insidious onset and slow progression (years) (Golbe, 2014; Levin et al., 2016; Höglinger et al., 2017)	Rapid progression (months) (Tan et al., 2005; Adams et al., 2011; Dash et al., 2016; Takkar et al., 2020)	Rapid progression (months) or long disease duration (up to 18 years) (Gaig et al., 2017; Hierro et al., 2020)	Sudden onset and rapid, gradual progression (Josephs et al., 2002; Lanza et al., 2014)	Rapid progression (Magherini et al., 2007)
Cerebrospinal fluid	No established markers (Höglinger et al., 2017)	Possible: -elevated protein (Tan et al., 2005; Adams et al., 2011; Ohyagi et al., 2017) -elevated IgG (Adams et al., 2011) -pleocytosis (lymphocyte↑) (Tan et al., 2005; Dash et al., 2016) -onconeuronal antibodies (Dash et al., 2016; Simard et al., 2020)	Possible: -normal or inflammatory (Hierro et al., 2020; González-Ávila et al., 2021)	Limited data	-Polymerase chain reaction (+) for Tropheryma whipplei (Höglinger et al., 2017) - elevated IgG, (+) VDRL test (Veneral Diseases Research Laboratory) (Murialdo et al., 2000)
MRI	Midbrain atrophy (Höglinger et al., 2017)	Normal or uncharacteristic changes without midbrain atrophy (Tan et al., 2005; Adams et al., 2011; Dash et al., 2016; Ohyagi et al., 2017)	Limited data	Ischaemic changes in white matter and in basal ganglia (Josephs et al., 2002; Lanza et al., 2014)	Group of enhancing lesions/single mass, without midbrain atrophy (Panegyres et al., 2006; Peregrin and Malikova, 2015)
Additional symptoms associated with neoplastic disease	Limited data	Possible: -haemoglobin↓, cachaexia, fever (Tan et al., 2005) -history of neoplastic disease (Takkar et al., 2020) -paraneoplastic neurological symptoms, e.g., polyneuropathy (Tan et al., 2005)	Limited data	Limited data	Limited data
Risk factors of cerebrovascular disease	Hypertension (Rabadia et al., 2019)	Limited data	Limited data	Present (hypertension, diabetes, smoking, prior stroke, stenosis of carotid arteries) (Winikates and Jankovic, 1994; Lanza et al., 2014)	Limited data
Non-characteristic features in the clinical picture	Not applicable	Possible, e.g., -horizontal gaze palsy and anterocollitis (Takkar et al., 2020)	Possible, e.g., -response to immunotherapy (Hierro et al., 2020) -sleep disorders, facial dyskinesia (González-Ávila et al., 2021)	Possible, e.g., -pathologic reflexes, unilateral paresis, facial nerve palsy (Josephs et al., 2002) -loss of bladder control, asymmetric symptoms involving mainly the lower body (Winikates and Jankovic, 1994)	Possible, e.g., -response to antibiotics, pupillary dysfunction (Murialdo et al., 2000) -gastrointestinal symptoms, lymphadenopathy, weight loss, oculomasticatory myrhythmia (Magherini et al., 2007) -lack of square wave jerks, more impaired upward than downward saccades (Averbuch-Heller et al., 1999)

## Vascular Progressive Supranuclear Palsy

Vascular PSP is a rare syndrome associated with multiple ischemic lesions in various brain regions, including the brainstem, the basal ganglia, thalamus, frontal lobe, and cerebellum, without tau deposits characteristic of neurodegenerative PSP (Josephs et al., 2002). Clinical signs usually include falls, dementia, and other symptoms that mimic tau-related PSP, but a thorough physical examination additionally reveals atypical features (shown in Table 1) (Winikates and Jankovic, 1994; Josephs et al., 2002; Lanza et al., 2014). The key element of medical evaluation among this group of patients seems to be the assessment of cardiovascular risk factors and the demonstration of ischemic changes on imaging studies (Winikates and Jankovic, 1994; Josephs et al., 2002; Lanza et al., 2014).

## Progressive Supranuclear Palsy Associated With Infectious and Parasitic Diseases

Progressive supranuclear palsy-like syndrome is a rare presentation of bacterial diseases, including Whipple disease and neurosyphilis, both with potential response to antibiotics (Murialdo et al., 2000; Magherini et al., 2007). Neurological manifestations in Whipple’s disease usually do not appear until the advanced stages of the disease; however, in some cases, neurological disorders are the first and only symptoms of the disease (Gerard et al., 2002; Magherini et al., 2007). In contrast to neurodegenerative PSP, neurological examination reveals the lack of square wave jerks, more impaired upward than downward saccades, and oculomasticatory myrhythmia in some cases (Averbuch-Heller et al., 1999; Magherini et al., 2007). Imaging studies can show varied changes, but without midbrain atrophy characteristic of tau-related PSP (Panegyres et al., 2006; Peregrin and Malikova, 2015). In neurosyphilis, the presence of pupillary dysfunction is an important feature that may help in the differential diagnosis with tau- related PSP (Murialdo et al., 2000). Single cases of PSP-like syndrome in the course of viral infections (HIV virus) (Jang et al., 2011) and parasitic diseases (neurocysticercosis) (Sharma et al., 2011) are also described in the literature, but data about them are very limited.

The differential features of paraneoplastic, autoimmune, vascular, infectious, and neurodegenerative PSP are given in the Table 1.

## Conclusion

This work highlights the clinical diversity of PSP subtypes. The lack of optimal tools to make a precise ante-mortem diagnosis of rare PSP variants, especially in the early stages, still remains a major difficulty for clinicians. The development of neuroimaging methods, mainly nuclear imaging techniques, gives clinicians a chance to change the standards of patient management. To date, most reports have been related to differences in 18-FDG, 18F-flortaucipir and 18F-THK5351 uptake in PET across PSP variants, but new radiotracers are also emerging, such as [18F]PM-PBB3 (Amtage et al., 2014; Brendel et al., 2018; Pardini et al., 2019; Hsu et al., 2020; Whitwell et al., 2020; Ishizuchi et al., 2021). Determination of specific uptake patterns of individual markers is likely to make a breakthrough in the diagnostics of rare variants of PSP in cases where this is difficult or impossible on the basis of clinical features. The problem concerning the exclusion of PSP-like syndromes seems to be particularly important due to the overlapping of symptoms, the lack of consistent criteria, and biomarkers. Precise diagnosis could potentially be used to include patients in clinical trials for new PSP therapies (Boxer et al., 2017), and it is also important from the point of view of implementing treatment for reversible causes of PSP syndromes, since it offers a chance to improve the prognosis and even to reduce the neurological deficit in some cases. Further research is needed to find suitable *in vivo* “biomarkers” for the different variants of the disease. Nevertheless, this work allows clinicians to accurately target the medical examination and the anamnesis, which currently still remain the basis for the diagnosis of the rare variants of this atypical Parkinsonism.

## Author Contributions

PK involved in the study design, data analysis, review of the literature, writing – original draft preparation, and writing – review and editing. NM, AM, BM, and DJ involved in the review and editing. PA involved in the study design, writing – review and editing, supervision, and final acceptance. All authors contributed to the article and approved the submitted version.

## Conflict of Interest

The authors declare that the research was conducted in the absence of any commercial or financial relationships that could be construed as a potential conflict of interest.

## Publisher’s Note

All claims expressed in this article are solely those of the authors and do not necessarily represent those of their affiliated organizations, or those of the publisher, the editors and the reviewers. Any product that may be evaluated in this article, or claim that may be made by its manufacturer, is not guaranteed or endorsed by the publisher.
